# Supplementary material for: A UMLS-based spell checker for natural language processing in vaccine safety
Source: BMC Med Inform Decis Mak. 2007 Feb 12;7:3. doi: 10.1186/1472-6947-7-3 (PMC1805499; doi:10.1186/1472-6947-7-3)
Supplement: Additional file 13 — RAP application directory. Sets up directory for RAP (RDF application for PHP) source code [file 1472-6947-7-3-S13.gz › rap/doc/phpdoc/__filesource/fsource_utility__utilObject.php.html]

xml version="1.0" encoding="iso-8859-1"?


File Source for Object.php


# Source for file Object.php

Documentation is available at Object.php

```
1. <?php

3. // ----------------------------------------------------------------------------------
4. // Class: Object
5. // ----------------------------------------------------------------------------------


9. /**
10. * An abstract object.
11. * Root object with some general methods, that should be overloaded.
12. *
13. * <BR><BR>
14. * History:<UL>
15. * <li>09-10-2002 : First version of this class.</li>
16. * </UL>
17. *
18. *
19. * @version  V0.9.1
20. * @author Chris Bizer <chris@bizer.de>
21. *
22. * @abstract
23. * @package utility
24. *
25. */
26. class Object {

28. /**
29. * Serializes a object into a string
30. *
31. * @access    public
32. * @return    string
33. */
34. function toString() {
35. $objectvars = get_object_vars($this);
36. foreach($objectvars as $key => $value)
37. $content .= $key ."='". $value. "'; ";
38. return "Instance of " . get_class($this) ."; Properties: ". $content;
39. }

41. }


44. ?>
```

Documentation generated on Thu, 7 Jul 2005 13:42:18 +0200 by phpDocumentor 1.3.0RC3
